# Implementing Optimal Care Pathways for Aboriginal and Torres Strait Islander People With Cancer: A Survey of Rural Health Professionals’ Self-Rated Learning Needs

**DOI:** 10.5334/ijic.6028

**Published:** 2022-03-30

**Authors:** Eli Ristevski, Teralynn Ludwick, Michael Leach, Sharyn Thompson, Mahesh Iddawela, Michelle Pryce, Elaine Wood, Kerry Davidson, Joanne Gell

**Affiliations:** 1 Monash University, School of Rural Health, Warragul, Victoria, AU; 2 University of Melbourne, Melbourne School of Population and Global Health, Victoria, AU; 3 Monash University, School of Rural Health, Bendigo, Victoria, AU; 4 School of Rural Health, Warragul, Victoria, AU; 5 Latrobe Regional Hospital, Traralgon, Victoria, Australia; 6 Alfred Health, Department of Medical Oncology, Melbourne, Victoria, AU; 7 Gippsland Regional Integrated Cancer Services, Traralgon, Victoria, AU; 8 Grampians Integrated Cancer Service, Ballarat, Victoria, AU

**Keywords:** integrated care, optimal care pathways, cancer, clinician, cultural safety, Aboriginal, Torres Strait Islander

## Abstract

**Objective:**

In 2018, the Optimal Care Pathway (OCP) for Aboriginal and Torres Strait Islander people with cancer was developed in Australia to improve the cancer care experiences and outcomes of Aboriginal and Torres Strait Islander people.

**Methods:**

Our study examined health professionals’ learning needs to meet the clinical practice requirements of the new OCP. An electronic questionnaire was distributed to 120 health professionals providing oncology care in two rural areas in Victoria, Australia. Questions included demographics, practice, cancer OCPs and implementation recommendations. Descriptive, chi-square and thematic analyses were undertaken.

**Results:**

Fifty-two health professionals from medicine (21%), nursing (37%) and allied health (37%) responded. All OCP sub-categories were selected, with a mean of 23 sub-categories identified as areas requiring additional learning. Aboriginal and Torres Strait Islander Perspectives, Treatment, and End of Life were the categories of higher interest. Care After Initial Treatment and Recovery was the category of lower interest. For respondents without cultural training, sub-categories involving practical tasks were of significant interest. Cultural education, connecting with Aboriginal and Torres Strait Islander services, putting learning into practice and respect emerged as themes.

**Conclusion:**

Strategies to address gaps included cultural safety training, person and family centred practice, and partnerships and connections with Aboriginal and Torres Strait Islander people and organisations across primary and tertiary sectors.

## Introduction

Indigenous peoples across the world experience significantly lower cancer survival outcomes and poorer experiences of healthcare than non-Indigenous peoples [1,2]. In 2018, cancer authorities in Australia introduced the Optimal Care Pathway (OCP) for Aboriginal and Torres Strait Islander people with cancer to address the significantly lower cancer outcomes among this group, and in recognition that many existing cancer care services fail to provide accessible and culturally appropriate care [3,4,5]. Designed to be used in conjunction with existing tumour-specific OCPs, this OCP provides guidance to health professionals and health services for culturally appropriate health care environments [6].

OCPs, also known as critical care pathways or integrated care pathways, are complex interventions that support mutual decision-making as well as organisation and standardisation of care to decrease fragmentation and variation in care [7,8]. The European Pathway Association states that OCPs should feature: alignment of care with best practice, patient expectations, and characteristics; facilitate communication between providers, patients, and families; define, sequence and coordinate care roles and activities; track outcomes; and identify appropriate resources [7,9]. Integrated cancer care supports a coordinated continuum of care over the course of the patient journey, from diagnosis and treatment through to palliative and end-of-life care [10]. From a patient perspective, integrated care enables cancer patients to work with care providers to achieve the outcomes of importance to them [11]. However, OCPs are often defined more narrowly with reference to discrete points in the pathway, focusing more on the sequencing of activities and delineation of professional roles than patient-centred elements [8,12]. In the Australian context, OCPs are currently available for 18 tumour types and map the care pathway with due consideration to current best practice, clinical guidelines, research and clinical consensus. Steps in the OCP span from prevention and early detection through to end-of-life care, with supportive care overarching all the steps. The Aboriginal and Torres Strait Islander OCP was developed as a companion document to the tumour-specific versions of OCPs [4].

The OCP for Aboriginal and Torres Strait Islander people with cancer extends the integrated care approach to recognise the importance of culture for Aboriginal and Torres Strait Islander people in their health care experiences and health outcomes [13,14,15]. It also recognised the ongoing experiences of discrimination and racism in health services [16,17], and access to culturally safe services as an essential requirement of person-centred care for Aboriginal and Torres Strait Islander people [18]. ‘Understanding your patient’, ‘communication’, and ‘practical considerations for consultations’ are underlying elements of the OCP that traverse all the steps in the pathway and are foundational for delivering person-centred, integrated cancer care to Aboriginal and Torres Strait Islander people [19].

While the OCP has been nationally endorsed, its implementation relies on the capacity of health services and practitioners to incorporate it into clinical practice. To date, implementation of tumour-specific OCPs in Australia has focussed on clinical audits of operational, diagnostic and referral processes as outcome measures [20,21,22]. Cultural and psychosocial elements may not be readily documented in clinical notes or have defined or standardised categories for measurement. Furthermore, many studies identify gaps in health professionals’ education, knowledge and confidence to provide culturally appropriate care to Indigenous peoples [23,24,25]. As one of the first OCPs to be guided by principles of culturally safe practice, more information was required on how to support health professionals to effectively implement this OCP into clinical practice. Our study aimed to understand health professionals’ perspectives on the knowledge, communication, and clinical practice skills they required to implement the OCP for Aboriginal and Torres Strait Islander people with cancer into clinical practice. We examined the following questions:

1. What are health professionals’ self-rated learning needs to implement the OCPs for Aboriginal and Torres Strait Islander people with cancer into their practice?
2. What is the influence of prior cultural training on health professionals’ learning needs?
3. What recommendations do health professionals have to implement the OCP into practice?

Note, the term Indigenous is used when referenced in reports, studies or quotations.

## Methods

### Participants

A survey of health professionals was undertaken in two rural regions of Victoria, Australia. Stratified and convenience sampling was used to recruit health professionals from six public rural hospitals. Key staff from medicine (Medical Oncologist, Radiation Oncologist, Haematologist, Surgeon, Specialist Physician (e.g. respiratory)), nursing (Chemotherapy Nurse, Tumour specific Nurse (e.g. breast care nurse, prostate nurse), Nurse Practitioner/Candidate, Radiation Therapy Nurse) and allied health (Aboriginal Health/Liaison Worker, Dietitian, Occupational Therapist, Psychologist, Physiotherapist, Pharmacist, Radiation Therapist, Speech Pathologist, Social Worker), who provided direct care to cancer patients and worked regularly at the service (full or part-time) were eligible to participate. Staff in administration only roles and casual positions were excluded. ***Table 1*** illustrates the targeted recruitment numbers by hospital and reflects the available oncology workforce according to the inclusion criteria. Hospitals 3–6 had visiting medical oncology and haematology staff 1–2 days/week from Hospital 1. Not all services had the full range of nursing and allied health specialties. Each region had access to one public radiation therapy service which operated five days per week. The availability of chemotherapy services varied between one and five days per week, with a capacity of 8–110 patients weekly. Oncology care was provided to 1,378–1,740 new cancer cases per year, across a population size of 220,000–283,039 per region.

**Table 1:** Targeted recruitment numbers by hospital.


SITE (HOSPITAL PEER GROUP CLASSIFICATION)	REMOTENESS AREA	MEDICINE	NURSING	ALLIED HEALTH	TOTAL (n)

Hospital 1 Public acute Group A	Inner regional	8	12	12	32

Hospital 2 Public acute Group A	Inner regional	8	12	12	32

Hospital 3 Public Acute Group A	Inner regional	0	10	8	20

Hospital 4 Public acute Group B	Inner regional	0	10	8	20

Hospital 5 Public Acute Group C	Inner regional	0	5	5	10

Hospital 6 Public Acute Group C	Outer regional	0	5	5	10


### Questionnaire

An electronic link to the ‘Optimal Care Pathway Clinician Survey’ was sent to the hospital Directors and Managers for dissemination to potential participants; a paper questionnaire could also be completed on request. Participants were also recruited through a multidisciplinary tumour meeting. The survey was distributed between 24 November – 23 December 2019 (region 1) and 12 February 19 March 2020 region 2). An explanatory statement was included in the questionnaire. Completing the questionnaire indicated consent to participate and was anonymous to complete. This study was approved by the Monash University Human Research Ethics Committee (approval number: 13621).

The Optimal Care Pathway Clinician Survey’ was a 47-item questionnaire developed by the researchers. Sections included: demographic (n = 4), clinical practice (n = 5), OCPs (n = 36) and two open-ended questions: Any other comments about providing care to Aboriginal and Torres Strait Islander cancer patients and families/carers and Do you have any recommendations about how we can implement the Aboriginal and Torres Strait Islander Optimal Care Pathway in your health service? The questionnaire items were based on key actions within each step of the OCP for Aboriginal and Torres Strait Islander people with cancer [3] as well as hospital accreditation safety and quality standards relevant to the care of Aboriginal and Torres Strait Islander people in Australia [26].

Questions on the OCP for Aboriginal and Torres Strait Islander people with cancer were divided into nine categories:

1. Prevention and early detection
2. Presentation, initial investigations and referral
3. Diagnosis, staging & treatment planning
4. Treatment
5. Care after initial treatment and recovery
6. Supportive care
7. Recurrent, residual and metastatic disease
8. End of life
9. Aboriginal and/or Torres Strait Islander perspectives

Categories 1–8 reflected the seven key steps and overarching Supportive Care component in the OCP. We created the ninth category entitled ‘Aboriginal and/or Torres Strait Islander Perspectives’. This category consolidated the OCP preamble describing Aboriginal and Torres Strait Islander peoples’ connection with “land, culture, community and identity” and a “whole-of-life view” of health (OCP ref, pg 1).

Within each of these nine categories, we created questions (sub-categories) asking participants to select which OCP sub-categories they would like to know more about to provide care to Aboriginal and Torres Strait Islander people with cancer. Multiple sub-categories could be selected (***Table 2***).

**Table 2:** Optimal Care Pathway questions by category and sub-category.


CATEGORY 1: ABORIGINAL AND/OR TORRES STRAIT ISLANDER PERSPECTIVES ON	

**Sub-categories**	Health, illness, well-being.

	Cancer perspectives (meaning, fears, concerns, and taboos).

	Gender-specific matters (‘Men’s Business’ and ‘Women’s Business’).

	The connection between country, spirituality, family, community and health.

	Spiritual practices, traditional healers, and traditional, complementary or alternative medicine therapies.

	Knowing when to use traditional terminology (e.g. when to use ‘Aunty’ or ‘Uncle’).

**CATEGORY 2: PREVENTION AND EARLY DETECTION: USING CULTURALLY APPROPRIATE INFORMATION TO DISCUSS**	

**Sub-categories**	Risk reduction (e.g. quit smoking and healthy eating).

	Screening and immunisation (e.g. mammograms and HPV vaccination).

	Early detection (cancer signs and symptoms, and co-morbidities).

**CATEGORY 3: PRESENTATION, INITIAL INVESTIGATIONS AND REFERRAL)**	

**Sub-categories**	Using culturally relevant information to explain the reasons for diagnostic/referral investigations to the patient and their family/carer.

	Addressing patient and family concerns about cancer and cancer treatment.

**CATEGORY 4: DIAGNOSIS, STAGING & TREATMENT PLANNING**	

**Sub-categories**	Understanding factors which influence Aboriginal and/or Torres Strait Islander patients’ decisions about treatment and ongoing care.

	Speaking in a culturally appropriate way about treatment options and the expected outcomes of these treatments.

	Checking/knowing if the person has understood the information I have provided about the treatment plan.

	Access to an expert with culturally appropriate knowledge in the multidisciplinary meetings (MDM).

	Culturally appropriate resources to discuss and seek informed consent to participate in clinical trials (if clinically appropriate).

**CATEGORY 5: TREATMENT**	

**Sub-categories**	Practising trauma-informed care using culturally informed approaches.

	Understanding cultural practices in the clinical setting (e.g. touching patients and who to discuss diagnosis/prognosis with).

	Working with families during cancer treatment and follow-up care.

	Understanding cultural perceptions about pain experiences, relief and management.

	Pathways/processes to work with the Aboriginal Hospital Liaison Officer/Aboriginal Health Worker during treatment and follow-up care.

	Knowing about Indigenous-specific patient assistance programs/schemes (e.g. Close the Gap prescriptions).

	Understanding the potential barriers to Aboriginal and/or Torres Strait Islander people accessing treatment, health services, and/or follow-up care.

**CATEGORY 6: CARE AFTER INITIAL TREATMENT AND RECOVERY**	

**Sub-categories**	Developing culturally appropriate treatment summaries and/or follow-up care plans.

	Strategies to provide culturally appropriate information about the signs and symptoms of recurrent and secondary prevention of disease.

	Strategies to provide culturally appropriate information about healthy living after cancer treatment.

	Information about referral options/pathways for social and emotional well-being and mental health services.

	Processes to keep a patient’s General Practitioner updated (e.g. prognosis and a follow-up care plan).

**CATEGORY 7: SUPPORTIVE CARE**	

**Sub-categories**	Using the Supportive Care Need Assessment Tool - Indigenous Patients (SCNAT-IP) to identify supportive care needs.

	Using a culturally appropriate pain tool to better identify and manage pain.

	Culturally appropriate supportive care services (internal and external to service).

**Category 8: RECURRENT, RESIDUAL AND METASTATIC DISEASE**	

**Sub-categories**	Using culturally appropriate language to explain treatment intent, outcomes or adverse events for recurrent, residual or metastatic disease.

	Discussing advance care planning in a culturally relevant manner with patients and their families/carers.

	Discussing referral to palliative care with patients and their families/carers.

**CATEGORY 9: END OF LIFE CARE**	

**Sub-categories**	Using culturally appropriate language when discussing death or dying.

	Discussing cultural preferences related to practices around death and dying.


The questionnaire was tested for face and content validity among ten people, including those with experience in literacy and plain language, those who work with Aboriginal people and Aboriginal Community Controlled Organisations, allied health professionals (dietetics and radiation therapy), oncology nurses, medical practitioners (haematologists and medical oncologists) and those who work in Indigenous research and policy.

This study was funded under the Victorian Government Cancer Survivorship Program Grants and through in-kind support from Gippsland Regional Integrated Cancer Services and Grampians Integrated Cancer Service.

### Data analysis

Descriptive analyses of demographic variables, clinical practice variables, and OCP categories/sub-categories were undertaken. All categorical variables were summarised in terms of the frequency and percentage. The continuous variables ‘Clinical experience (years)’ and ‘Number of sub-categories each participant wanted to know more about’ were summarised using the median as well as the minimum, lower quartile (25^th^ percentile), upper quartile (75^th^ percentile) and maximum. Across all OCP categories combined, the percentages of respondents who wanted to know more about each OCP sub-category were sorted in order of decreasing magnitude ahead of division into two groups: high interest (third and fourth quartiles) and low interest (first and second quartiles). In order to visualise these data using a bar chart, the percentages for each OCP sub-category were then further stratified by OCP category. For each sub-category of the OCP survey, a two-way cross-tabulation of cultural awareness training (yes/no) versus interest in knowing more (yes/no) was generated. Pearson’s chi-square test was used to assess the statistical significance of differences in which categories those with and without cultural awareness training identified as learning needs. An available case approach was used to deal with missing data. P-values<0.05 indicated statistical significance. All statistical analyses were undertaken using Microsoft Excel or IBM SPSS Statistics for Windows, Version 25.0 (Armonk, NY: IBM Corp.).

Thematic analysis was used to analyse the open-ended questions [27]. Thematic analysis was appropriate to our exploratory question to understand participant perspectives and ideas on learning needs, gaps and suggestions for implementation into practice. The Principal Investigator (ER) reviewed the responses to the open-ended questions and grouped the participants’ words and phrases to form concepts. The data were further examined to identify if concepts could be merged or expanded, or whether new concepts needed to be created. This process resulted in the formation of provisional themes. The research team reviewed the provisional themes to identify relationships, make comparisons, and note contrasting or emerging themes. The process was complete when there was consensus among the researchers that no new themes could be identified.

## Results

### Demographic and practice variables

A total of 52/120 health professionals (response rate = 43%) from medicine (21%), nursing (37%) and allied health (37%) responded to the survey. The diversity of professions included: medicine (medical oncology, radiation oncology, surgery, and haematology), nursing (chemotherapy, haematology, palliative care, radiation nursing, and tumour-specific specialist nursing), and allied health (Aboriginal Health Liaison Officer [AHLO], dietetics, occupational therapy, pharmacy, physiotherapy, radiation therapy, social work, and speech pathology). The mean years of clinical experience was 16 years, with the main areas of clinical practice being treatment and supportive care. The majority of participants (96%) did not identify as Aboriginal and/or Torres Strait Islander people. A total of 79% of participants reported providing care to Aboriginal and/or Torres Strait Islander people, 46% had attended cultural awareness training in the last five years, 50% reported they were always confident asking patients if they identify as Aboriginal and/or Torres Strait Islander people, and 23% were aware of the OCP for Aboriginal and Torres Strait Islander people with cancer (***Table 3***).

**Table 3:** Demographic and practice variables for health professionals who responded to the questionnaire.


VARIABLE (N = 52)^†^	n	%

**Clinical experience (years)**		

Mean (Minimum-Maximum)	16 (1–41)	

**Profession:**		

Nursing	19	36.5%

Allied Health	19	36.5%

Medical	11	21.2%

Missing	3	5.8%

**Identify as Aboriginal and/or Torres Strait Islander**		

Yes, Aboriginal	2	3.8%

Neither Aboriginal nor Torres Strait Islander	50	96.2%

**Provide services/care in^#^:**		

Treatment	41	78.8%

Supportive care	29	55.8%

Care after initial treatment and recovery	24	46.2%

Managing recurrent, residual and metastatic disease	23	44.2%

Palliative care	22	42.3%

End-of-life care	16	30.8%

Diagnosis, staging and treatment planning	13	25.0%

Presentation, initial investigations and referral	9	17.3%

Prevention and early detection	5	9.6%

**Provide care to Aboriginal and Torres Strait Islander people:**		

Yes	41	78.8%

No	8	15.4%

Not sure	3	5.8%

**Attended cultural awareness/competency/safety training in the last 5 years:**		

Yes	24	46.2%

No	25	48.1%

Cannot remember	3	5.8%

**Confident in asking patients if they identify as Aboriginal and/or Torres Strait Islander:**		

Yes, always	26	50.0%

Sometimes	18	34.6%

No	5	9.6%

Not sure	3	5.8%

**Aware of cancer Optimal Care Pathway for Aboriginal and Torres Strait Islander people:**		

Yes	12	23.1%

No	34	65.4%

Not sure	6	11.5%


^†^ Unless otherwise specified. ^#^ More than one choice selected.

### Optimal care pathways

All the OCP sub-category were selected with a minimum and maximum of 0–36 sub-categories of interest, a median of 23, and 15 and 34 sub-categories in the 25^th^ and 75^th^ percentile, respectively.

**Figure 1:**
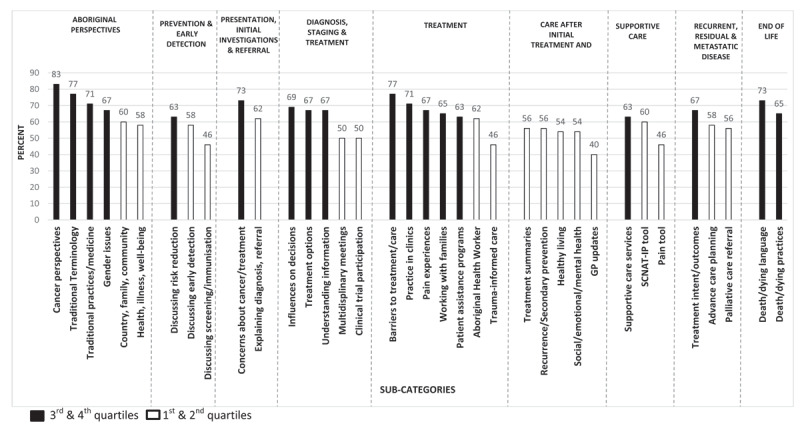
OCP sub-categories by high versus low interest (N = 52). GP = general practitioner; SCNAT-IP = Supportive Care Need Assessment Tool – Indigenous Patients.

Sub-categories of interest in the lower 1^st^ and 2^nd^ quartiles, were predominately from the ‘Care after initial treatment and recovery’ category (***Figure 1***). When ordered by percentage, the lowest sub-categories of interest included:

- 40% GP updates (Care after initial treatment & recovery)
- 46% Pain tool (Supportive care)
- 46% Trauma-informed care (Treatment)
- 46% Discussing screening/immunisation (Prevention and early detection)
- 50% Clinical trial participation (Diagnosis, staging & treatment planning)
- 50% MDMs (Diagnosis, staging & treatment planning)
- 54% Social/emotional/mental health (Care after initial treatment & recovery)
- 54% Healthy living (Care after initial treatment & recovery).

Sub-categories of most interest in the higher 3^rd^ and 4^th^ quartiles were predominately from the ‘Aboriginal perspectives’, ‘Treatment’ and ‘End of Life’ categories (***Figure 1***). When ordered by percentage, the sub-categories of highest interest included:

- 83% Cancer perspectives (Aboriginal perspectives)
- 77% Traditional Terminology (Aboriginal perspectives)
- 77% Barriers to treatment/care (Treatment)
- 73% Concerns about cancer/treatment (Presentation, initial investigation & referral)
- 73% Death/dying language (End of life)
- 71% Practice in clinics (Treatment)
- 71% Traditional practices/medicine (Aboriginal perspectives)
- 69% Influences on decisions (Diagnosis, staging and treatment planning).

### Readiness for practice

Chi-square tests of responses by participants who had cultural training compared to those without cultural training showed statistically significant differences for seven sub-categories. Significantly higher percentages of participants without cultural training were found among those who wanted to know more about: practicing trauma-informed care, advance care planning, referral to palliative care, clinical trial participation, cultural knowledge in MDMs, patient assistance programs, and cultural practices in clinical settings (see Supporting table S1).

### Open-ended responses

Twenty participants provided responses to the open-ended questions. The demographics of the participants who responded to the opened-end questions mirrored that of the overall sample. The following themes were identified as recommendations for how to implement the OCP into the health service. Quotations are used to illustrate themes and reported verbatim as written by participants.

#### Cultural education

Providing cultural education about Aboriginal and Torres Strait Islander people emerged as an overarching theme. Education session should be flexible in terms of timing and delivery mode to allow all staff to attend outside of clinic times. Training should be mandatory to engage staff and form part of intern/trainee orientation. Updates should also be offered regularly rather than sporadically. Conducting a general awareness session on how to access the OCP was also suggested.

“The short awareness session that I attended served to show me how much I do not know. Providing more of these opportunities with education on respectful communication and treatment are essential to better care” (020Nursing).

Training and education should be delivered by and/or involve Aboriginal and Torres Strait Islander people from the local community.

“Using our own ATSI [*sic*] community to conduct workshops perhaps off the back of a grand round- maybe this should/could happen each year” (010Medical).

#### Information

There were suggestions for further information on two topics: process and practical issues as well as demographic and statistical information. Participants stated they wanted more information on how to access medication prescription programs, the role of the AHLO, and inclusive family practices in discussing treatment, death, and dying.

“Clear documentation on how to implement services- i.e. closing the gap for prescriptions. Internal process can be unclear” (004Allied Health).

Participants also wanted more demographic and statistical information such as cancer statistics and outcomes for Aboriginal and Torres Strait Islander people, in general, and in the local area.

“Provide data on how many Aboriginal & Torres Strait Islander patients came through for cancer care and what tumour sites - this will engage the relevant clinicians” (048Medical).

#### Connecting with the local community

Connecting with the Aboriginal community in the local area and Aboriginal health and community services outside of the hospital setting could also support clinicians’ practice. Promoting cultural ceremonies and events was also suggested as a strategy to learn more about and connect with Aboriginal and Torres Strait Islander people.

“Use the local Aboriginal services in your local towns as these people will be the ones helping the person in between appointments” (022AlliedHealth).

However, some reservations also emerged.

#### Putting into practice

Comments arose regarding the low number of Aboriginal and Torres Strait Islander people presenting for care. Participants stated that it is not always possible to put learning into practice and that information could be forgotten if not used regularly.

“There are low numbers of patients presenting with Cancer from an ATSI [*sic*] background in this region. A generic focus on health literacy and some information about cultural safety might be useful but possible that it be forgotten again without having opportunity to implement concepts learned through education” (005Noprofession).

#### Understanding and Respect

There were also reservations about the appropriateness and value of the support they could offer to Aboriginal people as a non-Aboriginal person, particularly as past practices have not been culturally appropriate towards Aboriginal and/or Torres Strait Islander people.

“I think as a non-ATSI [*sic*] [Health Worker] without appropriate training I’m very wary that I’m unlikely to be of much benefit to an ATSI [*sic*}] Oncology patient, especially given historical context of ATSI [*sic*] “helping professions” interactions” [011AlliedHealth].

It was stated that “There is nothing more patronising than acting like we understand their situation when we don’t” (031Alliedhealth). People did not want their behaviours as a non-Aboriginal person to be seen as disrespectful: “You don’t want to be disrespectful” (050Nursing). Countering this, there were comments about treating Aboriginal and Torres Strait Islander people “as you would like to be treated” (031AlliedHealth).

## Discussion

Our study found health professionals providing cancer care in two rural areas of Victoria felt underprepared to implement the OCP for Aboriginal and Torres Strait Islander people with cancer. Less than 50% of participants had cultural training or were confident in asking patients if they identified as an Aboriginal and/or Torres Strait Islander person. All nine categories and sub-categories in the OCP were of interest. Sub-categories of most interest focussed on disease, treatment and procedural practices. Sub-categories of least interest focussed on health promotion, well-being and primary care. Greater provision of cultural education, cancer statistics and connecting with the local Aboriginal community and health services were strategies recommended for implementing the OCP. There were also uncertainties about putting cultural learning into practice in settings with small numbers of Aboriginal and Torres Strait patients. Queries were also raised regarding whether non-Indigenous people could provide culturally appropriate care to Aboriginal and Torres Strait Islander people. Implementing the OCP into clinical practice as part of integrative care will require a multifaceted approach at the patient, health professional and health system levels. ***Table 4*** illustrates some strategies which can be used to implement an integrative approach to the optimal care pathway for Aboriginal and Torres Strait Islander people with cancer. The strategies reflect the key findings of our study, relevant policy documents [3,26,28,29,30] and the peer reviewed literature.

**Table 4:** An integrative approach to implementing Optimal Care Pathways for Aboriginal and Torres Strait Islander people with cancer.


ISSUES	PATIENT	HEALTH PROFESSIONAL	HEALTH SYSTEM

**CULTURAL SAFETY**	Use culturally appropriate methods to evaluate patient experiences of care.	Undertake and maintain cultural safety training. Use principles of cultural safety in clinical practice.	Implement regular and refresher cultural safety training. Partner with Elders and Aboriginal Community to ensure training is culturally safe. Evaluate organisational processes and practices in cultural safety.

**OPTIMAL CARE PATHWAYS**	Respect for Aboriginal and Torres Strait Islander culture, family, Community, Country	Use person-centred practice. Take a wholistic approach. Use culturally appropriate assessment tools. Connect with Aboriginal hospital, health & liaison workers. Include cultural supports in Multidisciplinary Meetings.	Create welcoming environments. Find out the needs of local Community. Use self-determination to respond to needs.

**CONNECTIONS AND PARTNERSHIPS**	Create supports for patients outside of hospital system.	Establish links with primary care (e.g. General Practitioners).	Connect with Elders and Community. Establish links with Aboriginal Community Controlled Health Organisations


### Cultural safety

In our study, cultural education emerged as a major gap in participants’ knowledge and skills and a key recommendation for implementing the OCP into clinical practice. We found it difficult to discern a stand out area within the OCP which required immediate action, as there was high interest in all the categories and sub-categories. We recommend health services move beyond general cultural awareness training by incorporating actions which upskill the cultural competency of the workforce and embed culturally safe practices into clinical practice and service delivery [31,32]. Systematic reviews show little evidence for the effectiveness of cultural awareness interventions beyond short-term effects on knowledge and confidence [33,34]. Cultural safety provides a foundation for understanding one’s own attitudes, beliefs, bias (conscious and unconscious), position, power and privilege. [35]. Cultural safety is also a continuum of learning from unaware, to emerging, capable and proficient [36]. In many countries, cultural safety is recognised as best practice [35,37] and an underlying foundation to integrative care and implementation strategies. Participants in our study wanted regular and refresher cultural training delivered by Aboriginal and Torres Strait Islander people and/or organisations. In recognition of the fact that Aboriginal and Torres Strait Islander people are not a homogenous group, health services could partner with Elders and Aboriginal and Torres Strait Islander organisations to ensure training is culturally safe and tailored to the context, needs and experiences of Aboriginal and Torres Strait Islander people in that community [31].

Participants’ concern about the low numbers of Aboriginal and Torres Strait Islander people presenting for care needs further investigation. This research was conducted in regions located within local government areas with high Aboriginal populations [38]. Furthermore, in the state of Victoria, cancer incidence and mortality are higher among Aboriginal and Torres Strait Islander people than non-Indigenous people [39]. While national health service standards for safety and quality in Australia require hospitals to collect and report on Indigenous status of patients [26], the collection is known to be incomplete [40]. Furthermore, Aboriginal and Torres Strait Islander people report they can feel unsafe to disclose their cultural identity for fear of discrimination and racism. Additionally, the processes for when, where and how these data are collected are inconsistent across health services. Specific training on how to ‘ask the question’ needs to be provided to all staff within the health service to ensure Aboriginal and Torres Strait Islander people feel safe accessing the health service, and the information is used in an appropriate and timely manner to refer patients to appropriate programs and services to aid in their cancer treatment and recovery.

Our study found there was interest in knowing more about, and connecting with, the local Aboriginal community and health services. Health services can use national quality and safety standards to create welcoming environments and processes to partner with consumers in the governance of the organisation [26]. Partnerships in governance, along with a self-determination approach, are more effective at identifying the needs of the community and ensuring programs and services are responsive to local needs [30].

Organisations also need to evaluate processes and practices in cultural safety against measures appropriate to Aboriginal and Torres Strait Islander people. Australia has numerous Indigenous-specific health service quality and safety standards, health performance frameworks and a cancer framework which can be used [17,26,29]. Efforts to improve the cultural safety of organisations and the workforce need to be evaluated through culturally appropriate patient-reported experience measures (PREMs) [15,41]. We recommended PREMs as they document processes such as interactions with staff, inclusive practices, respect, the provision of supportive care, follow-up care and care coordination, which remain as gaps in the service experiences of Aboriginal and Torres Strait Islander people [42,43].

### Optimal care pathways

While there are many areas of practice which require further learning, studies identify the areas of importance in cancer care for Aboriginal and Torres Strait Islander people are culture, the inclusion of family in their care, connections with community and Country, and practices free of discrimination and racism [13,15,44,45]. When cultural safety is coupled with a strength-based approach and person-centred practice, clinicians can get to know and understand the needs of the person and their family, the treatment outcomes desired and how they want their care to be provided. Putting the person and their family at the centre of care, rather than the disease, and taking a holistic approach, rather than a process focussed approach, can help clinicians build rapport and trust with patients [13]. Recognising the care pathway may not always be linear, particularly as Aboriginal and Torres Strait Islander people have a higher rate of advanced disease and cancers with low prognostic outcomes (e.g. lung, liver, head and neck cancers)[39]. Furthermore, financial, geographic, accommodation, transport and family responsibilities are factors which can be barriers to accessing help and services for Aboriginal and Torres Strait Islander people [13,46].

Clinicians can use culturally appropriate assessment tools to help to initiate and enhance discussion around sensitive issues such as death, dying, pain management and psychosocial care. While our study showed less interest in using clinical tools to support practices such as advance care planning (58%), palliative care referral (56%), treatment summaries (56%) and pain tools (46%), clinical tools can assist patients and clinicians with the early identification of unmet needs, and provide appropriate and timely referrals. Clinical tools can also help clinicians to ask the right questions as Aboriginal and Torres Strait Islander people may not initiate discussion about problems they experience. For example, when dealing with pain, Aboriginal and Torres Strait Islander people may not always disclose pain due to feelings of shame and fear of racism [47]. Furthermore, Garvey, Thewes [48] reported that 80% of non-Indigenous health professionals found the use of a culturally appropriate supportive care screening tool useful to clinical practice and 90% would continue to use the tool.

Health professionals and health services can also create communication pathways and processes, as well as clear delineation of roles and responsibilities between clinicians and Aboriginal Health Workers to ensure better treatment access and navigation of the hospital system. Our survey found 62% of participants were interested in the role of the AHLO with additional comments in the responses to the open-ended question. The presence of cultural experts at MDMs was of greatest interest to clinicians without cultural training (65%). Cultural supports in MDMs can bring knowledge and experience to support cultural understanding for practitioners who may not be confident that they are providing culturally appropriate care [49]. While AHLOs should be part of the interprofessional team, the onus for providing culturally care should not rest solely with them. AHLO roles in hospitals are limited in number. Even in large metropolitan hospitals, there may be only one AHLO responsible for responding to all disease groups and service areas. Furthermore, AHLOs may not be registered health workers or practitioners and should not be placed in a position that requires them to work out of their scope of practice [49].

### Connection and Partnerships

While there was interest in connecting with local Aboriginal health and community services, there was low interest (40%) in ‘processes to keep a patient’s General Practitioner updated’. Connections to general practitioners (GPs) and primary care services are important avenues for supporting patients and family members outside of tertiary care and hospitals. A systematic review by Meiklejohn, Mimery [50] found GPs can be partners in the patient’s care throughout the whole of the OCP. While there is growing research on survivorship models of shared care between sectors (primary and tertiary care) and practitioners (GPs, oncologists and nurses), there is little research on these models with Aboriginal and Torres Strait Islander people [13,51]. Further research needs to be conducted on a whole-of-pathway approach between clinicians in tertiary services and GPs in primary care. This connection is especially important as many Aboriginal people’s main source of medical care is the local Aboriginal Community Controlled Health Organisation (ACCHO). ACCHOs have a large role in primary care and can assist in supporting patients and families in prevention and early detection of disease (screening), post-treatment care and surveillance of disease, and supportive care needs and practical assistance during treatment.

### Limitations

Our study is limited by the small sample size but strengthened by the inclusion of professionals from multiple professions. While we sampled professionals, who worked regularly and directly with cancer patients, there may be higher or different learning needs for part-time and casual staff. It is also unknown whether people who did not respond to the survey may have more or less learning needs. There may be practitioners who are culturally unsafe in their practice and not recognise this. Alternatively, there may be practitioners delivering best practice who could be mentors and leaders in the workplace. Our sample was limited to two rural areas of Victoria, Australia. Learning needs may be different in other rural or metropolitan areas of Victoria or States in Australia. However, research with health practitioners in other States indicates that this is a national issue [24,52]. As our questionnaire only asked participants to indicate a ‘yes’ if they wanted more knowledge, it is unclear whether knowledge was completely lacking or they wanted to enhance existing knowledge. During face and content validity testing of an earlier version of the questionnaire, participants rated themselves low (on a five-point rating scale) across almost all questions and stated they scaled their responses up to neutral.

### Conclusion

Our study contributes to the evidence base around health workforce readiness to implement the OCP for Aboriginal and Torres Strait Islander people with cancer. By investigating workforce knowledge and training needs, health services can target the necessary continuing professional development activities required to build the cultural competency of their staff. Health services can use this information to review existing processes, policies and practices to support implementation of the OCP into clinical practice and evaluate how their systems promote culturally safe environments for Aboriginal and Torres Strait Islander people. Finally, partnerships between practitioners, health services and primary and tertiary care sectors can facilitate an integrated approach to the care and services provided to Aboriginal and Torres Strait Islander people with cancer and their families, with a view to improving cancer care experiences and outcomes among Aboriginal and Torres Strait Islander people.

## Additional File

The additional file for this article can be found as follows:

10.5334/ijic.6028.s1 Supporting table S1. Category and sub-category of interest with and without cultural training.

## References

[1] Moore SP, Antoni S, Colquhoun A, Healy B, Ellison-Loschmann L, Potter JD, et al. Cancer incidence in indigenous people in Australia, New Zealand, Canada, and the USA: a comparative population-based study. The Lancet Oncology. 2015; 16(15): 1483–92. DOI: 10.1016/S1470-2045(15)00232-6 26476758

[2] Sarfati D, Robson B. Equitable cancer control: better data needed for indigenous people. The Lancet Oncology. 2015; 16(15): 1442–4. DOI: 10.1016/S1470-2045(15)00295-8 26476759

[3] Cancer Australia. Optimal care pathway for Aboriginal and Torres Strait Islander people with cancer. 2018. DOI: 10.1200/jgo.18.97700

[4] Chynoweth J, Daveson B, McCambridge M, Coutts J, Zorbas H, Whitfield, et al. A National Priority: Improving Outcomes for Aboriginal and Torres Strait Islander People With Cancer Through an Optimal Care Pathway. Journal of Global Oncology. 2018; 4(2): 243s–s.

[5] Australian Institute of Health Welfare. Cancer in Australia 2019. Canberra: AIHW; 2019.

[6] Chynoweth J, McCambridge MM, Zorbas HM, Elston JK, Thomas RJS, Glasson WJH, et al. Optimal Cancer Care for Aboriginal and Torres Strait Islander People: A Shared Approach to System Level Change. JCO Glob Oncol. 2020(6): 108–14. DOI: 10.1200/JGO.19.00076 PMC6998013 32031448

[7] Seys D, Panella M, VanZelm R, Sermeus W, Aeyels D, Bruyneel L, et al. Care pathways are complex interventions in complex systems: New European Pathway Association framework. International Journal of Care Coordination. 2019; 22(1): 5–9. DOI: 10.1177/2053434519839195

[8] Otty Z, Brown A, Sabesan S, Evans R, Larkins S. Optimal Care Pathways for People with Lung Cancer- a Scoping Review of the Literature. Int J Integr Care. 2020; 20(3): 14. DOI: 10.5334/ijic.5438 PMC7528692 33041731

[9] European Pathway Association. E-P-A Care Pathways. Available from: http://e-p-a.org/care-pathways/.

[10] Bergin RJ, Whitfield K, White V, Milne RL, Emery JD, Boltong A, et al. Optimal care pathways: A national policy to improve quality of cancer care and address inequalities in cancer outcomes. Journal of Cancer Policy. 2020; 25: 100245. DOI: 10.1016/j.jcpo.2020.100245

[11] National Voices. Principles of integrated care. London: National Voices; 2012.

[12] Mur-Veeman I, Hardy B, Steenbergen M, Wistow G. Development of integrated care in England and the Netherlands: Managing across public–private boundaries. Health Policy. 2003; 65(3): 227–41. DOI: 10.1016/S0168-8510(02)00215-4 12941491

[13] Ristevski E, Thompson S, Kingaby S, Nightingale C, Iddawela M. Understanding Aboriginal Peoples’ Cultural and Family Connections Can Help Inform the Development of Culturally Appropriate Cancer Survivorship Models of Care. JCO Glob Oncol. 2020; 6: 124–32. DOI: 10.1200/JGO.19.00109 32031446 PMC6998014

[14] Meiklejohn JA, Bailie R, Adams J, Garvey G, Bernardes CM, Williamson D, et al. “I’m a Survivor”: Aboriginal and Torres Strait Islander Cancer Survivors’ Perspectives of Cancer Survivorship. Cancer Nurs. 2020; 43(2): 105–14. DOI: 10.1097/NCC.0000000000000671 30543569

[15] Green M, Cunningham J, Anderson K, Griffiths K, Garvey G. Measuring health care experiences that matter to Indigenous people in Australia with cancer: identifying critical gaps in existing tools. Int J Equity Health. 2021; 20(1): 100. DOI: 10.1186/s12939-021-01433-2 33845852 PMC8042987

[16] Australian Insitute of Health and Welfare. Australia’s welfare 2017. Canberra: AIHW; 2017. Report No.: Australia’s welfare series no. 13. AUS 214.

[17] Australian Insitute of Health and Welfare. Aboriginal and Torres Strait Islander Health Performance Framework 2020 summary report. Cat. no. IHPF 2. Canberra: AIHW; 2020.

[18] Green M, Cunningham J, O’Connell D, Garvey G. Improving outcomes for Aboriginal and Torres Strait Islander people with cancer requires a systematic approach to understanding patients’ experiences of care. Australian health review: a publication of the Australian Hospital Association. 2017; 41(2): 231–3. DOI: 10.1071/AH15214 27385494

[19] de Witt A, Matthews V, Bailie R, Garvey G, Valery PC, Adams J, et al. Communication, Collaboration and Care Coordination: The Three-Point Guide to Cancer Care Provision for Aboriginal and Torres Strait Islander Australians. Int J Integr Care. 2020; 20(2): 10. DOI: 10.5334/ijic.5456 PMC7292184 32565760

[20] Bergin RJ, Thomas RJS, Whitfield K, White V. Concordance between Optimal Care Pathways and colorectal cancer care: Identifying opportunities to improve quality and reduce disparities. Journal of Evaluation in Clinical Practice. 2020; 26(3): 918–26. DOI: 10.1111/jep.13231 31287616

[21] Kabwe M, Robinson A, Shethia Y, Parker C, Blum R, Solo I, et al. Timeliness of cancer care in a regional Victorian health service: A comparison of high-volume (Lung) and low-volume (oesophagogastric) tumour streams. Cancer Reports. 2021; 4(1): e1301. DOI: 10.1002/cnr2.1301 33026194 PMC7941434

[22] Rogers MJ, Garrard B, Kress R, Kim M, Cameron H, Matheson L, et al. Optimal care pathways for lung cancer in South West Victoria. Australian Journal of Cancer Nursing, The. 2019; 20(2): 4–7. DOI: 10.33235/ajcn.20.2.4-7

[23] Meiklejohn JA, Adams J, Valery PC, Walpole ET, Martin JH, Williams HM, et al. Health professional’s perspectives of the barriers and enablers to cancer care for Indigenous Australians. European Journal of Cancer Care. 2016; 25(2): 254–61. DOI: 10.1111/ecc.12467 26918690

[24] Taylor EV, Haigh MM, Shahid S, Garvey G, Cunningham J, Holloway M, et al. Australian cancer services: a survey of providers’ efforts to meet the needs of Indigenous patients. Australian and New Zealand Journal of Public Health. 2018; 42(6): 547–52. DOI: 10.1111/1753-6405.12843 30370959

[25] Garvey G, Cunningham J, Mayer C, Letendre A, Shaw J, Anderson K, et al. Psychosocial Aspects of Delivering Cancer Care to Indigenous People: An Overview. JCO Glob Oncol. 2020(6): 148–54. DOI: 10.1200/JGO.19.00130 PMC6998016 32031444

[26] The Wardliparingga Aboriginal Research Unit of the South Australian Health and Medical Research Institute. National Safety and Quality Health Service Standards user guide for Aboriginal and Torres Strait Islander health. Sydney: Australian Commission on Safety and Quality in Health Care; 2017.

[27] Taylor SJ, Bogdan R. Introduction to qualitative research methods: A guidebook and resource. New York: John Wiley & Sons, Inc; 1998.

[28] Cancer Australia. A guide to implmenting the optimal care pathway for Aboriginal and Torres Strait Islander people with cancer. Strawberry Hills, NSW: Cancer Australia; 2020.

[29] Cancer Australia. National Aboriginal and Torres Strait Islander Cancer Framework. Surry Hills, NSW; 2015.

[30] Victorian Government. Korin Korin Balit-Djak: Aboriginal health, well-being and safety strategic plan 2017–2027. Melbourne; 2017.

[31] Bainbridge R, McCalman J, Clifford A, Tsey K. Cultural competency in the delivery of health services for Indigenous people. Issues paper no. 13. Produced for the Closing the Gap Clearinghouse. Canberra; 2015.

[32] Shepherd SM. Cultural awareness workshops: limitations and practical consequences. BMC Medical Education. 2019; 19(1): 14. DOI: 10.1186/s12909-018-1450-5 30621665 PMC6325797

[33] Horvat L, Horey D, Romios P, Kis-Rigo J. Cultural competence education for health professionals. Cochrane Database Syst Rev. 2014(5): Cd009405. DOI: 10.1002/14651858.CD009405.pub2 PMC10680054 24793445

[34] Jongen C, McCalman J, Bainbridge R. Health workforce cultural competency interventions: a systematic scoping review. BMC Health Services Research. 2018; 18(1): 232. DOI: 10.1186/s12913-018-3001-5 29609614 PMC5879833

[35] National Collaborating Centre for Indigenous Health. Towards Cultural Safety for Métis: An Introduction for Heath Care Providers. Canada: University of Northern British Columbia.; 2013.

[36] Services DoHaH. Aboriginal and Torres Strait Islander cultural safety framework - cultural safety continuum reflective tool - Part 2. Melbourne; 2019.

[37] Nursing Council of New Zealand. Guidelines for cultural safety, the treaty of Waitangi, and Maori health in nursing and midwifery education and practice. Wellington: Nursing Council of New Zealand; 2011.

[38] Victorian Public Sector Commission. Aboriginal Cultural Capability Toolkit 2019. [updated 28 June 2019]. Available from: https://vpsc.vic.gov.au/html-resources/aboriginal-cultural-capability-toolkit/.

[39] Victorian Cancer Registry. Cancer in Victoria: Statistics & Trends 2019. Melbourne, Victoria; 2020.

[40] Griffiths K, Coleman C, Al-Yaman F, Cunningham J, Garvey G, Whop L, et al. The identification of Aboriginal and Torres Strait Islander people in official statistics and other data: Critical issues of international significance. Statistical Journal of the IAOS. 2019; 35(1): 91–106. DOI: 10.3233/SJI-180491

[41] Thurber KA, Walker J, Batterham PJ, Gee GC, Chapman J, Priest N, et al. Developing and validating measures of self-reported everyday and healthcare discrimination for Aboriginal and Torres Strait Islander adults. International Journal for Equity in Health. 2021; 20(1): 14. DOI: 10.1186/s12939-020-01351-9 33407521 PMC7788827

[42] Reilly R, Micklem J, Yerrell P, Banham D, Morey K, Stajic J, et al. Aboriginal experiences of cancer and care coordination: Lessons from the Cancer Data and Aboriginal Disparities (CanDAD) narratives. Health Expect. 2018; 21(5): 927–36. DOI: 10.1111/hex.12687 29691974 PMC6186541

[43] Garvey G, Cunningham J, Janda M, Yf He V, Valery PC. Psychological distress among Indigenous Australian cancer survivors. Support Care Cancer. 2018; 26(6): 1737–46. DOI: 10.1007/s00520-017-3995-y 29243167

[44] Meiklejohn JA, Bailie R, Adams J, Garvey G, Bernardes CM, Williamson D, et al. “I’m a Survivor”: Aboriginal and Torres Strait Islander Cancer Survivors’ Perspectives of Cancer Survivorship. Cancer Nurs. 2018. DOI: 10.1097/NCC.0000000000000671 30543569

[45] Butler TL, Anderson K, Garvey G, Cunningham J, Ratcliffe J, Tong A, et al. Aboriginal and Torres Strait Islander people’s domains of wellbeing: A comprehensive literature review. Social science & medicine (1982). 2019; 233: 138–57. DOI: 10.1016/j.socscimed.2019.06.004 31200269

[46] Anderson K, Diaz A, Parikh DR, Garvey G. Accessibility of cancer treatment services for Indigenous Australians in the Northern Territory: perspectives of patients and care providers. BMC Health Services Research. 2021; 21(1): 95. DOI: 10.1186/s12913-021-06066-3 33509170 PMC7841038

[47] Strong J, Nielsen M, Williams M, Huggins J, Sussex R. Quiet about pain: experiences of Aboriginal people in two rural communities. Aust J Rural Health. 2015; 23(3): 181–4. DOI: 10.1111/ajr.12185 25945587

[48] Garvey G, Thewes B, He V, Davis E, Girgis A, Valery P, et al. Indigenous cancer patient and staff attitudes towards unmet needs screening using the SCNAT-IP. Support Care Cancer. 2016; 24(1): 215–23. DOI: 10.1007/s00520-015-2770-1 26003424

[49] Taylor EV, Lyford M, Parsons L, Mason T, Sabesan S, Thompson SC. “We’re very much part of the team here”: A culture of respect for Indigenous health workforce transforms Indigenous health care. PLOS ONE. 2020; 15(9): e0239207. DOI: 10.1371/journal.pone.0239207 32960933 PMC7508383

[50] Meiklejohn JA, Mimery A, Martin JH, Bailie R, Garvey G, Walpole ET, et al. The role of the GP in follow-up cancer care: a systematic literature review. J Cancer Surviv. 2016; 10(6): 990–1011. DOI: 10.1007/s11764-016-0545-4 27138994

[51] Meiklejohn JA, Arley B, Bailie R, Adams J, Garvey G, Martin JH, et al. Community-identified recommendations to enhance cancer survivorship for Aboriginal and Torres Strait Islander people. Aust J Prim Health. 2018; 24(3): 233–40. DOI: 10.1071/PY17127 29804561

[52] Croager EJ, Eades T, Pratt IS, Slevin T. Impact of a short, culturally relevant training course on cancer knowledge and confidence in Western Australia’s Aboriginal Health Professionals. Aust N Z J Public Health. 2010; 34 Suppl 1: S76–9. DOI: 10.1111/j.1753-6405.2010.00558.x 20618300

