# Supplementary material for: Implementing Optimal Care Pathways for Aboriginal and Torres Strait Islander People With Cancer: A Survey of Rural Health Professionals’ Self-Rated Learning Needs
Source: Int J Integr Care. 2022 Mar 30;22(1):27. doi: 10.5334/ijic.6028 (PMC8973837; doi:10.5334/ijic.6028)
Supplement: Supporting table S1. — Category and sub-category of interest with and without cultural training. [file ijic-22-1-6028-s1.pdf]

Supporting table S1: Category and sub-category of interest with and without cultural training

	Cultural Training		
**Category and Sub-Category of Optimal Care Pathway Survey**	**No n (row %)**	**Yes n (row %)**	**Sig.***
**ABORIGINAL AND/OR TORRES STRAIT ISLANDER PERSPECTIVES ON:**			
Health, illness, well-being.			No
No, I would not like to know more	9 (47.4%)	10 (52.6%)	
Yes, I would like to know more	16 (53.3%)	14 (46.7%)	
Cancer (meaning, fears, concerns, taboos).			N/A
No, I would not like to know more	4 (57.1%)	3 (42.9%)	
Yes, I would like to know more	21 (50.0%)	21 (50.0%)	
Gender-specific matters ('Men's Business' and 'Women's Business').			N/A
No, I would not like to know more	2 (66.7%)	1 (33.3%)	
Yes, I would like to know more	23 (50.0%)	23 (50.0%)	
The connection between country, spirituality, family, community and health.			N/A
No, I would not like to know more	1 (50.0%)	1 (50.0%)	
Yes, I would like to know more	24 (51.1%)	23 (48.9%)	
Spiritual practices, traditional healers, traditional, complementary or alternative medicine therapies.			N/A
No, I would not like to know more	1 (50.0%)	1 (50.0%)	
Yes, I would like to know more	24 (51.1%)	23 (48.9%)	
Knowing when to use traditional terminology (e.g. when to use 'Aunty' or 'Uncle').			N/A
No, I would not like to know more	6 (60.0%)	4 (40.0%)	
Yes, I would like to know more	19 (48.7%)	20 (51.3%)	
**PREVENTION AND EARLY DETECTION**			
Risk reduction (e.g. quit smoking, healthy eating)			No
No, I would not like to know more	5 (31.3%)	11 (68.8%)	
Yes, I would like to know more	20 (60.6%)	13 (39.4%)	
Screening and immunisation (e.g. mammograms, HPV vaccination)			No
No, I would not like to know more	11 (42.3%)	15 (57.7%)	
Yes, I would like to know more	14 (60.9%)	9 (39.1%)	
Early detection (cancer signs and symptoms, co-morbidities)			No
No, I would not like to know more	8 (38.1%)	13 (61.9%)	
Yes, I would like to know more	17 (60.7%)	11 (39.3%)	
**PRESENTATION, INITIAL INVESTIGATIONS AND REFERRAL**			
Using culturally relevant information to explain the reasons for diagnostic investigations to the patient and their family/carer.			No
No, I would not like to know more	7 (36.8%)	12 (63.2%)	

Yes, I would like to know more	18 (60.0%)	12 (40.0%)	
Addressing patient and family **concerns about cancer** and cancer treatment			No
No, I would not like to know more	4 (33.3%)	8 (66.7%)	
Yes, I would like to know more	21 (56.8%)	16 (43.2%)	
**DIAGNOSIS, STAGING & TREATMENT PLANNING**			
Understanding factors which influence Aboriginal and/or Torres Strait Islander patients' **decisions** about treatment and on-going care.			No
No, I would not like to know more	6 (42.9%)	8 (57.1%)	
Yes, I would like to know more	19 (54.3%)	16 (45.7%)	
Speaking in a culturally appropriate way about **treatment options** and the expected outcomes of these treatments.			No
No, I would not like to know more	6 (37.5%)	10 (62.5%)	
Yes, I would like to know more	19 (57.6%)	14 (42.4%)	
Checking/knowing if the person has **understood the information** I have provided about the treatment plan.			No
No, I would not like to know more	5 (33.3%)	10 (66.7%)	
Yes, I would like to know more	20 (58.8%)	14 (41.2%)	
Access to an expert with culturally appropriate knowledge in the Multidisciplinary Meetings (**MDM**).			Yes
No, I would not like to know more	8 (34.8%)	15 (65.2%)	
Yes, I would like to know more	17 (65.4%)	9 (34.6%)	
Culturally appropriate resources to discuss and seek informed consent to participate in **clinical trials** (if clinically appropriate)			Yes
No, I would not like to know more	8 (34.8%)	15 (65.2%)	
Yes, I would like to know more	17 (65.4%)	9 (34.6%)	
**TREATMENT**			
Practising **trauma-informed care** using culturally informed approaches			Yes
No, I would not like to know more	9 (36.0%)	16 (64.0%)	
Yes, I would like to know more	16 (66.7%)	8 (33.3%)	
Understanding **cultural practices** in the clinical setting e.g. touching patients, who to discuss diagnosis/prognosis with			Yes
No, I would not like to know more	4 (28.6%)	10 (71.4%)	
Yes, I would like to know more	21 (60.0%)	14 (40.0%)	
Working with **families** during cancer treatment and follow-up care			No
No, I would not like to know more	6 (37.5%)	10 (62.5%)	
Yes, I would like to know more	19 (57.6%)	14 (42.4%)	
Understanding cultural perceptions about **pain** experiences, relief and management.			No

No, I would not like to know more	6 (40.0%)	9 (60.0%)	
Yes, I would like to know more	19 (55.9%)	15 (44.1%)	
Pathways/processes to work with the **Aboriginal Hospital Liaison Officer/ Aboriginal Health Worke**r during treatment and follow-up care.			No
No, I would not like to know more	7 (41.2%)	10 (58.8%)	
Yes, I would like to know more	18 (56.3%)	14 (43.8%)	
Knowing about Indigenous-specific **patient assistance programs**/schemes (e.g. close the gap prescriptions).			Yes
No, I would not like to know more	5 (27.8%)	13 (72.2%)	
Yes, I would like to know more	20 (64.5%)	11 (35.5%)	
Understanding the **potential barriers** for Aboriginal and/or Torres Strait Islander people in accessing treatment, health services, and/or follow-up care.			N/A
No, I would not like to know more	4 (44.4%)	5 (55.6%)	
Yes, I would like to know more	21 (52.5%)	19 (47.5%)	
**CARE AFTER INITIAL TREATMENT AND RECOVERY**			
Developing culturally appropriate **treatment summaries** and/or follow-up care plans.			No
No, I would not like to know more	8 (36.4%)	14 (63.6%)	
Yes, I would like to know more	17 (63.0%)	10 (37.0%)	
Strategies to provide culturally appropriate information about the signs and symptoms of **recurrent and secondary prevention** of disease.			No
No, I would not like to know more	8 (38.1%)	13 (61.9%)	
Yes, I would like to know more	17 (60.7%)	11 (39.3%)	
Strategies to provide culturally appropriate information about **healthy living** after cancer treatment.			No
No, I would not like to know more	9 (40.9%)	13 (59.1%)	
Yes, I would like to know more	16 (59.3%)	11 (40.7%)	
Information about referral options/pathways for **social and emotional well-being** and **mental health services**.			No
No, I would not like to know more	8 (38.1%)	13 (61.9%)	
Yes, I would like to know more	17 (60.7%)	11 (39.3%)	
Processes to keep a patient's **General Practitioner** updated (e.g. prognosis, follow-up care plan).			No
No, I would not like to know more	11 (39.3%)	17 (60.7%)	
Yes, I would like to know more	14 (66.7%)	7 (33.3%)	
**SUPPORTIVE CARE**			
Using the Supportive Care Need Assessment Tool - Indigenous Patients (SCNAT-IP) to identify **supportive care needs.**			No
No, I would not like to know more	8 (42.1%)	11 (57.9%)	

Yes, I would like to know more	17 (56.7%)	13 (43.3%)	
Using a culturally appropriate **pain tool** to better identify and manage pain.			No
No, I would not like to know more	11 (40.7%)	16 (59.3%)	
Yes, I would like to know more	14 (63.6%)	8 (36.4%)	
Culturally appropriate supportive care **services** (internal and external to service).			No
No, I would not like to know more	7 (43.8%)	9 (56.3%)	
Yes, I would like to know more	18 (54.5%)	15 (45.5%)	
**RECURRENT, RESIDUAL AND METASTATIC DISEASE**			
Using culturally appropriate language to **explain treatment intent, outcomes or adverse events** for recurrent, residual or metastatic disease.			No
No, I would not like to know more	7 (43.8%)	9 (56.3%)	
Yes, I would like to know more	18 (54.5%)	15 (45.5%)	
Discussing **Advance Care Planning** in a culturally relevant manner with patients and family/carers.			Yes
No, I would not like to know more	6 (30.0%)	14 (70.0%)	
Yes, I would like to know more	19 (65.5%)	10 (34.5%)	
Discussing referral to **palliative ca**re with patients and/families.			Yes
No, I would not like to know more	6 (30.0%)	14 (70.0%)	
Yes, I would like to know more	19 (65.5%)	10 (34.5%)	
**END OF LIFE CARE**			
Using culturally appropriate language when discussing death or dying.			No
No, I would not like to know more	6 (46.2%)	7 (53.8%)	
Yes, I would like to know more	19 (52.8%)	17 (47.2%)	
Discussing cultural preferences related to practices around death and dying			No
No, I would not like to know more	8 (50.0%)	8 (50.0%)	
Yes, I would like to know more	17 (51.5%)	16 (48.5%)	

HPV, human papillomavirus
